# Enantioselective nucleophilic difluoromethylation of aromatic aldehydes with Me_3_SiCF_2_SO_2_Ph and PhSO_2_CF_2_H reagents catalyzed by chiral quaternary ammonium salts

**DOI:** 10.3762/bjoc.4.21

**Published:** 2008-06-26

**Authors:** Chuanfa Ni, Fang Wang, Jinbo Hu

**Affiliations:** 1 Key Laboratory of Organofluorine Chemistry, Shanghai Institute of Organic Chemistry, Chinese Academy of Sciences, 354 Fenglin Road, Shanghai 200032, China

## Abstract

**Background:**

Although the nucleophilic difluoromethylation of aldehydes, ketones, and imines has been realized with PhSO_2_CF_2_H and related reagents, there are still no reports on the enantioselective nucleophilic reactions.

**Results:**

With a chiral quaternary ammonium salt as the catalyst and KOH as the base, we describe the first enantioselective difluoromethylation of aromatic aldehydes with PhSO_2_CF_2_H or Me_3_SiCF_2_SO_2_Ph. The enantioselectivity is substrate-dependent and for 2-chlorinated benzaldehyde an ee up to 64% was obtained.

**Conclusion:**

These results provide some insights into the enantioselective nucleophilic difluoromethylation chemistry, which will stimulate further progress in this field.

## Background

Because of the unique properties of fluorine, selective introduction of fluorine atom(s) or fluorine-containing moieties into organic molecules often dramatically alter their stability, lipophilicity, bioavailability, and biopotency. It is estimated that as many as 30–40% of agrochemicals and 20% of pharmaceuticals on the market contain fluorine [1–2]; as a result, fluorine is highlighted as the second most utilized hetero-element (after nitrogen) in life science-oriented research [3]. Nucleophilic fluoroalkylation, typically involving the transfer of a fluorinated carbanion (R_f_^−^, the fluorine substitution being commonly on the carbanion center) to an electrophile, represents one of the major methods for the synthesis of organofluorine compounds [4–18]. In recent years, a few methods have been reported for the enantioselective introduction of a trifluoromethyl group into aldehydes and ketones with Ruppert-Prakash reagent, (trifluoromethyl)trimethylsilane, Me_3_SiCF_3_ and other reagents [19–29]. Although one-step nucleophilic difluoromethylation with R_3_SiCF_2_H is challenging regarding generality and efficiency [30], we found that both PhSO_2_CF_2_H and Me_3_SiCF_2_SO_2_Ph can be used as general and efficient nucleophilic difluoromethylation reagents [11–12,31]. We have successfully synthesized both α-difluoromethyl alcohols and β-difluoromethyl alcohols by using these two reagents [12,16,31]. Moreover, we have described a highly diastereoselective nucleophilic difluoromethylation method with *N*-(*tert*-butylsulfinyl)imines using PhSO_2_CF_2_H as a difluoromethylation reagent [13]. However, enantioselective introduction of the difluoromethyl group has not been previously reported.

As a class of versatile catalysts for asymmetric synthesis, cinchona alkaloids and their derivatives can catalyze an amazing array of synthetically important reactions, providing access to chiral products of high enantiopurity [32–33]. Several examples have been reported on the enantioselective incorporation of trifluoromethyl [23–28] or monofluoromethyl group [34] into organic molecules catalyzed by chiral quaternary ammonium salts. In the course of our research on the fluoride ion-induced difluoromethylation of carbonyl compounds with Me_3_SiCF_2_SO_2_Ph, we found that the counterion of the fluoride source played an important role in tuning the fluoroalkylation reactivity towards aldehydes and ketones, i.e. aldehydes showed higher reactivity than ketones [12]. In connection with our studies on selective fluoroalkylation reactions, herein we disclose the first example of catalytic enantioselective difluoromethylation reaction of aromatic aldehydes with Me_3_SiCF_2_SO_2_Ph or PhSO_2_CF_2_H in the presence of a cinchona alkaloid-based chiral quaternary ammonium salt.

## Results and Discussion

### Enantioselective nucleophilic difluoromethylation of aromatic aldehydes with Me_3_SiCF_2_SO_2_Ph

We started our investigation with the reaction between benzaldehyde and Me_3_SiCF_2_SO_2_Ph, choosing the fluoride salt **4** as the initiator with 5 mol% loading (Table 1, entry 1). Catalyst **4** was reported to be a relatively effective catalyst in the reaction between Me_3_SiCF_3_ and aromatic aldehyde [23]. When the reaction was carried out in THF solvent at −78 °C, the reaction proceeded smoothly to afford the desired product in 91% yield with poor enantioselectivity (Table 1, entry 1). After a quick survey of the reaction solvent, the enantioselectivity was improved when toluene was used as solvent (Table 1, entries 1–4). It turned out that the least polar solvent (toluene) was an appropriate solvent for the reaction, while the use of polar solvents such as THF, Et_2_O and CH_2_Cl_2_ gave the product with relatively lower enantioselectivity. The increase of the catalyst loading was able to improve the enantioselectivity further. When 10 mol% of **4** was used, the ee was improved to 47% (Table 1, entry 5). However, when 2-naphthaldehyde or 4-methoxybenzaldehyde was used as the substrate, in each case lower ee was observed (Table 1, entries 6 and 7). Although catalyst **4** was ineffective in the catalysis of the nucleophilic difluoromethylation of ketones in toluene, the reaction could proceed smoothly when CH_2_Cl_2_ was added as a co-solvent. The tetrasubstituted difluoromethylated aryl alcohol was formed in 97% yield with 10% ee (Table 1, entry 8).

**Table 1:** Asymmetric nucleophilic difluoromethylation of aromatic aldehyde with PhSO_2_CF_2_SiMe_3_.

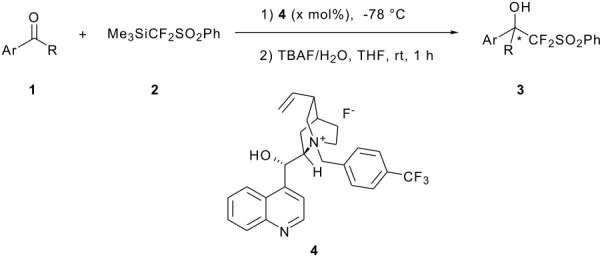					
entry^[a]^	carbonyl compound	Initiator [mol%]	solvent	yield [%]^[b]^	ee [%]^[c]^

1	PhCHO	**4** (5)	THF	91	14
2	PhCHO	**4** (5)	CH_2_Cl_2_	64	9
3	PhCHO	**4** (5)	Et_2_O	67	27
4	PhCHO	**4** (5)	PhCH_3_	65	36
5	PhCHO	**4** (10)	PhCH_3_	60	47
6	2-NapCHO	**4** (10)	PhCH_3_	64	26
7	*p*-MeOC_6_H_4_CHO	**4** (10)	PhCH_3_	50	25
8^[d]^	PhCOCH_3_	**4** (10)	PhCH_3_-CH_2_Cl_2_ (2:1, v/v)	97	10

[a] Unless noted, reactions were carried out at 1.0 mmol scale. To a mixture of **1** (1.0 equiv) and **4** (x mol%) in 2 mL of toluene at −78 °C, **2** (1.2 equiv, dissolved in 2 mL of toluene) was added dropwise over 2 hours. The reaction mixture was then stirred at the same temperature for additional 3 hours. [b] Isolated yield of the pure product. [c] Enantiomeric excess was determined by HPLC analysis using a chiral column. [d] The reaction was carried out at 0.25 mmol scale. **2** (4.0 equiv) in 0.5 mL PhCH_3_-CH_2_Cl_2_ was added in 5 minutes to the reaction mixture of **1** and **4** at −78 °C. The mixture was then stirred at the same temperature for another 12 hours.

### Enantioselective nucleophilic difluoromethylation of aromatic aldehydes with PhSO_2_CF_2_H

**Scheme 1:**
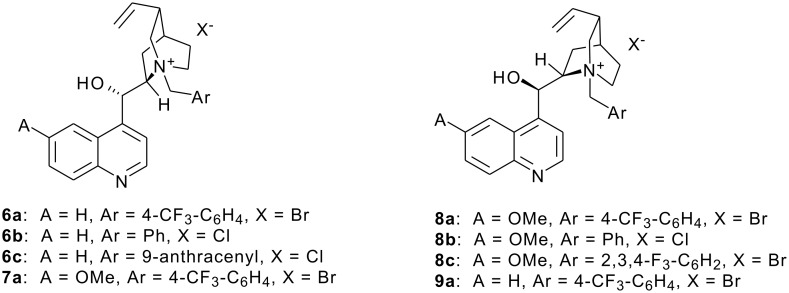
Structure of chiral quaternary ammonium salts.

In 1989, Stahly reported the nucleophilic difluoromethylation of aromatic aldehydes with PhSO_2_CF_2_H under the phase transfer condition using Aliquat 336 (a commercially available quaternary ammonium salt) as the phase transfer catalyst [35]. With most aldehydes, the reaction affords moderate to excellent yields of products. Moreover, unlike the Me_3_SiCF_2_SO_2_Ph-based reactions, the reaction with PhSO_2_CF_2_H is not water-sensitive [34]. The use of PhSO_2_CF_2_H as a robust fluoroalkylating agent [11] has aroused our interest in developing its application in enantioselective difluoromethylation reactions. Thus, we decided to evaluate the ability of known chiral ammonium salts to promote the enantioselective difluoromethylation of aldehydes with PhSO_2_CF_2_H. In a preliminary study, we examined the reaction with the chiral quaternary ammonium salt **6a** (see Scheme 1) as the phase transfer catalyst at room temperature, with 30% NaOH as the base and toluene as the solvent. After 12 h, the reaction afforded the desired product in 93% yield with a modest but significant enantioselectivity of 22% (Table 2, entry 1). When solid powdered KOH was used as the base, the ee could be slightly improved (Table 2, entry 2). Better enantioselectivity (47% ee) was observed on lowering the reaction temperature to −40 °C (Table 2, entry 3). However, at −78 °C, the reaction did not proceed.

**Table 2:** Enantioselective difluoromethylation of benzaldehyde with PhSO_2_CF_2_H under various conditions.

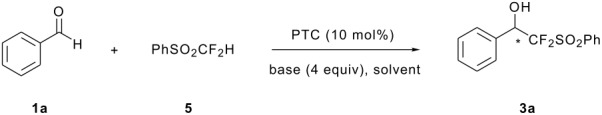							
Entry^[a]^	PTC	base	solvent	temperature [°C]	time [h]	yield [%]^[b]^	ee [%]^[c]^

1	**6a**	NaOH (30%)	PhCH_3_	RT	12	93	22
2	**6a**	solid KOH	PhCH_3_	RT	2	93	32
**3**	**6a**	**solid KOH**	**PhCH_3_**	−**40**	**48**	**67**	**47**
4	**7a**	solid KOH	PhCH_3_	−40	48	87	29
**5**	**8a**	**solid KOH**	**PhCH_3_**	**RT**	**2**	**94**	**46**
6	**8a**	solid KOH	PhCH_3_	−40	48	55	11
7	**9a**	solid KOH	PhCH_3_	RT	2	91	41
8	**8a**	CsOH(H_2_O)	PhCH_3_	RT	12	81	20
9	**8a**	RbOH (50%)	PhCH_3_	RT	12	76	2
10	**8a**	solid KOH	THF	RT	12	82	0
11	**8a**	solid KOH	CH_2_Cl_2_	RT	12	80	0
12	**6b**	solid KOH	PhCH_3_	−40	48	40	9
13	**6c**	solid KOH	PhCH_3_	−40	48	62	31
14	**8b**	solid KOH	PhCH_3_	RT	12	90	23
15	**8c**	RbOH (50%)	PhCH_3_	RT	12	0	–
16	**8c**	RbOH(H_2_O)	PhCH_3_	RT	12	80	5

[a] All reactions were carried out at 0.25 mmol scale with **1a** (1.2 equiv) and **5** (1.0 equiv) in 1.5 mL solvent. [b] Isolated yield of the pure product. [c] Enantiomeric excess was determined by HPLC analysis using a chiral column (Chiralcel AD-H eluting with 20% *i*-PrOH in hexane).

Using toluene as the solvent and solid KOH as the base, we scanned four 4-trifluoromethylphenyl ammonium salts derived from quinine (QN), quinidine (QD), cinchonine (CN), and cinchonidine (CD) at different reaction temperatures. We found the structure of the cinchona alkaloid had some influence on the enantioselectivity. When a cinchonine or quinidine derivative was used, the main isomer was obtained as (+)-**3a**, and CN **6a** was superior to QD **7a**. The optimized reaction temperature was −40 °C (Table 2, entries 2 and 3). For quinine derivative **8a**, it is interesting that a high temperature was beneficial for the enantioselectivity and (−)-**3a** was obtained as the main isomer. When the reaction was conducted at −40 °C, the reaction proceeded with moderate yield and poor enantioselectivity due to the low solubility of the catalyst in toluene (Table 2, entries 5 and 6). For CD derivative **9a**, the enantioselectivity was slightly lower than **8a** at rt (Table 2, entry 7). As reported, cinchonine derivatives and quinine derivatives yield products with the opposite configuration [32]. From the above screening, the quaternary salts **6a** and **8a** derived from the CN and QN were selected as the catalysts for further study.

Subsequently, the solvent effect was examined with catalyst **8a** in the presence of solid KOH as the base at room temperature. It was shown that the use of toluene as a reaction medium remarkably improved the enantioselectivity. When THF or CH_2_Cl_2_ was used, the complete loss of enantioselectivity was observed (Table 2, entries 10 and 11). Moreover, KOH gave the best results in enantioselectivity in comparison with the other metal hydroxides, such as CsOH and RbOH (Table 2, entries 8 and 9). Encouraged by these results, we further examined the influence of substituents on the chiral phase transfer catalysts. As shown in Table 2, the different substituents showed some influence on the enantioselectivity. The electron-withdrawing group CF_3_ at C-4 position of the benzyl ring afforded the product with good ee, though the unsubstituted one gave a significantly low ee (Table 2, entries 12 and 14). Although the 9-anthracenylmethyl and 2,3,4-trifluorobenzyl functionality were found to be quite useful for asymmetric alkylation of *tert*-butylglycinate Schiff base [36] or asymmetric synthesis of α,β-epoxysulfones [37], they were not as effective as 4-trifluoromethylphenyl functionality in the current asymmetric reaction (Table 2, entries 13, 15 and 16).

In the light of these results, we next examined the substrate scope of this enantioselective difluoromethylation reaction with catalyst **6a** or **8a**, and the results are shown in Table 3. Although the two types of cinchona alkaloids **6a** and **8a** are almost equally effective when benzaldehyde was tested, **6a** was chosen due to its generality towards other aldehydes such as 4-chlorobenzaldehyde **1b**. In general, the chemical yields were good to excellent, except in the case of 4-*tert*-butylbenzaldehyde **1k**, where a moderate yield was obtained (although the reaction was performed at rt) (Table 3, entry 13). It is obvious that the enantioselectivity was dependent on the substrate structure. It is interesting that the aldehydes with halogen substitution (Table 3, entries 3, 5–9) showed better enantioselectivity than those with methyl and methoxy substituents (Table 3, entries 11,12). Among the halogenated benzaldehydes that were tested, the reaction with 2-chlorobenzaldehyde showed an enantiomeric excess up to 64% (Table 3, entry 7). The enantiomeric excess obtained from 2-naphthaldehyde was also modest (23% ee) (Table 3, entry 14).

**Table 3:** Asymmetric nucleophilic difluoromethylation of aromatic aldehydes with PhSO_2_CF_2_H.

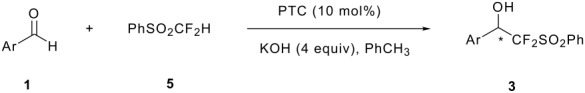						
entry^[a]^	aromatic aldehyde	PTC	T [°C]	time [h]	yield [%]^[b]^	ee [%]^[c]^

1	**1a**:Ar=C_6_H_5_-	**6a**	−40	48	**3a**: 67	47 (*S*)^[d]^
2	**1a**:Ar=C_6_H_5_-	**8a**	25	2	**3a**: 94	46 (*R*)^[e]^
3	**1b**:Ar=4-Cl-C_6_H_4_-	**6a**	−40	48	**3b**: 74	52 (*S*)^[f]^
4	**1b**:Ar=4-Cl-C_6_H_4_-	**8a**	25	1	**3b**: 91	23 (*R*)^[e]^
5	**1c**:Ar=2,4-Cl-C_6_H_3_-	**6a**	−20	48	**3c**: 95	54 (*S*)^[f]^
6	**1d**: Ar=3-Cl-C_6_H_4_-	**6a**	−20	48	**3d**: 83	46 (*S*)^[f]^
7	**1e**: Ar=2-Cl-C_6_H_4_-	**6a**	−20	48	**3e**: 92	64 (*S*)^[f]^
8	**1f**: Ar=4-F-C_6_H_4_-	**6a**	−20	48	**3f**: 93	41 (*S*)^[f]^
9	**1g**: Ar=4-Br-C_6_H_4_-	**6a**	−20	48	**3g**: 95	36 (*S*)^[f]^
10	**1h**:Ar=4-CF_3_-C_6_H_4_-	**6a**	−20	48	**3h**: 68	36 (*S*)^[f]^
11	**1i**: Ar=2-Me-C_6_H_4_-	**6a**	−20	48	**3i**: 77	11
12	**1j**:Ar=4-MeO-C_6_H_4_-	**6a**	−20	48	**3j**: 80	12
13	**1k**:Ar=4-*t*Bu-C_6_H_4_-	**6a**	25	12	**3k**: 58	4
14	**1l**: Ar=2-naphthyl-	**6a**	−20	48	**3l**: 72	23 (*S*)^[f]^

[a] All reactions were carried out at 0.25 mmol scale with **1** (1.2 equiv) and **5** (1.0 equiv) in 1.5 mL toluene. [b] Isolated yield of the pure product. [c] Enantiomeric excess was determined by HPLC analysis using a chiral column (Chiralcel AD-H, OD or IC). [d] The absolute configuration was determined to be *S* after chemical derivatization. [e] The absolute configuration was determined to be *R* by comparing the retention time on chiral HPLC. [f] The absolute stereochemistry was tentatively determined by ^19^F NMR analysis of the corresponding Mosher's esters comparing with (*S*)-**3a**.

**Scheme 2:**
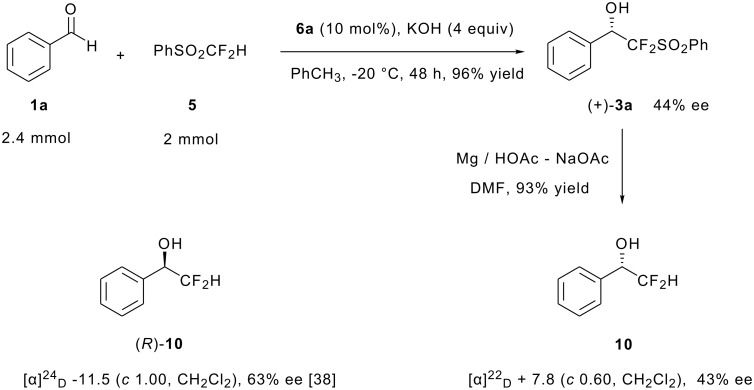
Determination of the absolute configuration of (+)-**3a**.

The absolute configuration of the alcohol (+)-**3a** (Table 3, entry 1) was determined to be *S* by comparing the optical rotation with that of the corresponding difluoromethyl alcohol (after desulfonylation) with the known data (Scheme 2) [38]. For other alcohols, the stereochemistry was tentatively determined by ^19^F NMR analysis of the corresponding Mosher's esters comparing with (+)-**3a** [39].

## Conclusion

In conclusion, we have described the first chiral quaternary ammonium salts catalyzed enantioselective difluoromethylation of aromatic aldehydes with Me_3_SiCF_2_SO_2_Ph and PhSO_2_CF_2_H. The enantioselectivity is substrate-dependent and for 2-chlorinated benzaldehydes, an ee up to 64% was obtained. The easy preparation of the chiral cinchonium salts and the convenient experimental procedure make the reaction operationally simple. These results provide some insights into enantioselective nucleophilic difluoromethylation chemistry, which will stimulate further progress in this field.

## Supporting Information

File 1 Full experimental details and compound characterization data for all new compounds described.
