# Supplementary material for: Enantioselective nucleophilic difluoromethylation of aromatic aldehydes with Me3SiCF2SO2Ph and PhSO2CF2H reagents catalyzed by chiral quaternary ammonium salts
Source: Beilstein J Org Chem. 2008 Jun 26;4:21. doi: 10.3762/bjoc.4.21 (PMC2486490; doi:10.3762/bjoc.4.21)
Supplement: File 1 — Full experimental details and compound characterization data for all new compounds described. [file Beilstein_J_Org_Chem-04-21-s001.doc]

**Enantioselective nucleophilic difluoromethylation of aromatic aldehydes with Me_3_SiCF_2_SO_2_Ph and PhSO_2_CF_2_H reagents catalyzed by chiral quaternary ammonium salts**

**Chuanfa Ni, Fang Wang and Jinbo Hu***

Key Laboratory of Organofluorine Chemistry, Shanghai Institute of Organic Chemistry, Chinese Academy of Sciences, 354 Fenglin Road, Shanghai 200032, China

[jinbohu@mail.sioc.ac.cn](mailto:jinbohu@mail.sioc.ac.cn)

**Supporting Information**

**1.1 Preparation of the chiral quaternary ammonium salts.**

The chiral quaternary ammonium salt **4** was prepared according to the reported procedure [1].

The chiral quaternary ammonium salts **6**–**8** were prepared from the corresponding cinchona alkaloids by refluxing with benzyl bromide or chloride in THF and filtration [1-4].

**2.1 General procedure for the asymmetric difluoromethylation reaction with PhSO_2_CF_2_H.**

**2.1.1. 2,2-Difluoro-1-phenyl-2-(phenylsulfonyl)ethanol (3a)** To a solution of benzaldehyde (**la**) (32 mg, 0.3 mmol), difluoromethyl phenyl sulfone (**5**) (48 mg, 0.25 mmol) and PTC **6a** (14 mg, 0.025 mmol) in toluene (1.5 mL) was added powdered KOH portion wise (56 mg, 1.0 mmol) at −40 °C. After the mixture was stirred at −40 °C for 48 h, the reaction mixture was quenched with 1N HCl (1.5 mL), and the mixture was extracted with ethyl acetate (10 mL X 3). The organic extracts were washed with brine and dried over MgSO_4_. Removal of the solvent followed by flash column chromatography of the residue (silica gel, hexane:AcOEt = 6:1) gave **4a** as a colorless solid (50 mg, 67%, 47% ee): [α]^22^_D_ 19.5 (*c* = 0.8, CHCl_3_); ee was determined by HPLC analysis: CHIRALPAK AD-H, hexane:*i*-PrOH = 80:20, flow rate 0.7 mL/min, *t*_major_ = 14.79 min, *t*_minor_ = 16.42 min.

**2.1.2. 1-(4-Chlorophenyl)-2,2-difluoro-2-(phenylsulfonyl)ethanol (3b)**

^1^H NMR (300 MHz, CDCl_3_): δ 3.75 (s, 1H); 5.58 (d, *J* = 21 Hz, 1H); 7.33 (d, *J* = 8.4 Hz, 2H); 7.41 (d, *J* = 8.4 Hz, 2H); 7.56–7.76 (m, 3H); 7.96 (d, 7.6 Hz, 2H). ^19^F NMR (287 MHz, CDCl_3_): –104.1 (d, *J* =237 Hz, 1F); –119.2 (dd, *J* = 237 Hz, 21 Hz, 1F). MS (EI, *m/z*): 332 (M^+^), 143, 141, 113, 78, 77, 51, 44. The data are consistent with the ones previously reported [5]. [α]^22^_D_ 23.7 (*c* = 1.05, CHCl_3_) (52% ee); ee was determined by HPLC analysis: CHIRALPAK OD, hexane:*i*-PrOH = 95:5, flow rate 0.7 mL/min, *t*_major_ = 33.41 min, *t*_minor_ = 39.03 min.

**2.1.3. 1-(2,4-Dichlorophenyl)-2,2-difluoro-2-(phenylsulfonyl)ethanol (3c)** White solid, mp 115–116 °C. [α]^22^_D_ 36.6 (*c* = 1.05, CHCl_3_) (54% ee); ee was determined by HPLC analysis: CHIRALPAK AD-H, hexane:*i*-PrOH = 90:10, flow rate 0.7 mL/min, *t*_minor_ = 26.88 min, *t*_major_ = 27.84 min. ^1^H NMR (300 MHz, CDCl_3_): δ 3.45 (d, *J* = 1.5 Hz, 1H), 6.14 (dd, *J* = 21.5, 3.5 Hz, 1H), 7.31 (dd, *J* = 8.4, 1.8 Hz, 1H), 7.40 (d, *J* = 2.0 Hz, 1H), 7.60–7.70 (m, 3H), 7.80 (t, *J* = 7.5 Hz, 1H), 8.03 (d, *J* = 8.0 Hz, 2H). ^19^F NMR (287 MHz, CDCl_3_): δ –104.5 (d, *J* = 240 Hz, 1F), –120.6 (dd, *J* = 240, 23 Hz, 1F). ^13^C NMR (75 MHz, CDCl_3_): δ 66.7 (dd, *J* = 26, 19.4 Hz), 120.0 (dd, *J* = 300, 288 Hz), 127.6, 129.2, 129.5, 130.4, 130.7, 130.8, 132.3, 134.5, 135.8, 136.0. Anal. Calcd. for C_14_H_10_Cl_2_F_2_O_3_S: C, 45.79; H, 2.74. Found: C, 45.1; H, 3.17. MS (ESI, *m*/*z*): 389.0 (M+Na^+^). IR (KBr): 3488, 1585, 1472, 1344, 1334, 1159, 1122, 1090, 1002 cm^-1^.

**2.1.4. 1-(3-Chlorophenyl)-2,2-difluoro-2-(phenylsulfonyl)ethanol (3d).** White solid, mp 107–108 °C. [α]^22^_D_ 15.7 (*c* = 0.95, CHCl_3_) (46% ee); ee was determined by HPLC analysis: CHIRALPAK AD-H, hexane:*i*-PrOH = 90:10, flow rate 0.7 mL/min, *t*_major_ = 24.32 min, *t*_minor_ = 29.59 min. ^1^H NMR (300 MHz, CDCl_3_): δ 3.46 (s, 1H), 5.54 (d, *J* = 21 Hz, 1H), 7.27–7.40 (m, 3H), 7.50 (s, 1H), 7.63 (t, *J* = 8.4 Hz, 2H), 7.74–7.82 (m, 1H), 8.00 (d, *J* = 7.3 Hz, 2H). ^19^F NMR (287 MHz, CDCl_3_): δ –104.3 (d, *J* = 228 Hz, 1F), –119.8 (dd, *J* = 228, 19.8 Hz, 1F). ^13^C NMR (75 MHz, CDCl_3_): δ 70.6 (dd, *J* = 26, 19 Hz), 120.0 (dd, *J* = 300, 288 Hz), 126.3, 128.2, 129.4, 129.6, 129.7, 130.7, 132.6, 134.4, 135.7, 135.8. Anal. Calcd. for C_14_H_11_ClF_2_O_3_S: C, 50.53; H, 3.33. Found: C, 50.84; H, 3.79. MS (ESI, *m*/*z*): 355.0 (M+Na^+^). IR (KBr): 3553, 1577, 1449, 1335, 1158, 1110 cm^-1^.

**2.1.5. 1-(2-Chlorophenyl)-2,2-difluoro-2-(phenylsulfonyl)ethanol (3e).** White solid, mp 104–105 °C. [α]^22^_D_ 46.2 (*c* = 1.0, CHCl_3_) (64% ee); ee was determined by HPLC analysis: CHIRALPAK AD-H, hexane:*i*-PrOH = 90:10, flow rate 0.7 mL/min, *t*_major_ = 28.61 min, *t*_minor_ = 31.69 min. ^1^H NMR (300 MHz, CDCl_3_): δ 3.42 (s, 1H), 6.20 (d, *J* = 22 Hz, 1H), 7.28–7.41 (m, 3H), 7.64 (t, *J* = 7.9 Hz, 2H), 7.69–7.74 (m, 1H), 7.79 (t, *J* = 7.7 Hz, 1H), 8.05 (d, *J* = 7.8 Hz, 2H). ^19^F NMR (287 MHz, CDCl_3_): δ –104.3 (d, *J* = 238 Hz, 1F), –120.6 (dd, *J* = 238, 22 Hz, 1F). ^13^C NMR (75 MHz, CDCl_3_): δ 67.0 (dd, *J* = 26, 19 Hz), 120.3 (dd, *J* = 300, 290 Hz), 127.2, 129.4, 129.8, 130.6, 130.7, 131.8, 132.5, 133.8, 135.7. Anal. Calcd. for C_14_H_11_ClF_2_O_3_S: C, 50.53; H, 3.33. Found: C, 50.72; H, 3.73. MS (ESI, *m*/*z*): 355.0 (M+Na^+^). IR (KBr): 3480, 1478, 1452, 1443, 1338, 1317, 1155, 1122 cm^-1^.

**2.1.6. 2,2-Difluoro-1-(4-fluorophenyl)-2-(phenylsulfonyl)ethanol (3f).** White solid, mp 99–100 °C. [α]^22^_D_ 16.4 (*c* = 0.95, CHCl_3_) (41% ee); ee was determined by HPLC analysis: CHIRALPAK AD-H, hexane:*i*-PrOH = 95:5, flow rate 0.7 mL/min, *t*_major_ = 49.67 min, *t*_minor_ = 54.77 min. ^1^H NMR (300 MHz, CDCl_3_): δ 3.40 (d, *J* = 3.5 Hz, 1H), 5.58 (dt, *J* = 21, 2.6 Hz, 1H), 7.08 (t, *J* = 8.4 Hz, 2H), 7.63 (t, *J* = 7.8 Hz, 2H), 7.78 (t, *J* = 7.4 Hz, 1H), 8.00 (d, *J* = 8.0 Hz, 2H). ^19^F NMR (287 MHz, CDCl_3_): δ –104.5 (d, *J* = 238 Hz, 1F), –112.1 (m, 1F), –120.0 (dd, *J* = 238, 20 Hz, 1F). ^13^C NMR (75 MHz, CDCl_3_): δ 70.6 (dd, *J* = 26, 19 Hz), 115.5 (d, *J* = 22 Hz), 120.2 (t, *J* = 295 Hz), 129.4, 129.5 (d, *J* = 2.8 Hz), 130.0 (d, *J* = 8.3 Hz), 130.6, 132.6, 135.7, 163.1 (d, *J* = 248 Hz). Anal. Calcd. for C_14_H_11_F_3_O_3_S: C, 53.16; H, 3.51. Found: C, 53.11; H, 3.57. MS (ESI, *m*/*z*): 355.0 (M+Na^+^). IR (KBr): 3547, 1607, 1449, 1338, 1232, 1159, 1113 cm^-1^.

**2.1.7.** **1-(4-Bromophenyl)-2,2-difluoro-2-(phenylsulfonyl)ethanol (3g).** White solid, mp 107–108 °C. [α]^22^_D_ 13.4 (*c* = 1,0, CHCl_3_) (36% ee); ee was determined by HPLC analysis: CHIRALPAK OD, hexane:*i*-PrOH = 98:2, flow rate 0.7 mL/min, *t*_major_ = 94.99 min, *t*_minor_ = 104.2 min. ^1^H NMR (300 MHz, CDCl_3_): δ 3.43 (d, *J* = 3.8 Hz, 1H), 5.55 (dt, *J* = 21, 3.2 Hz, 1H), 7.36 (d, *J* = 8.4 Hz, 2H), 7.52 (d, *J* = 8.4 Hz, 2H), 7.63 (t, *J* = 7.3 Hz, 2H), 7.78 (t, *J* = 7.3 Hz, 1H), 7.99 (d, *J* = 7.5 Hz, 2H). ^19^F NMR (287 MHz, CDCl_3_): δ –104.5 (d, *J* = 238 Hz, 1F), –119.8 (dd, *J* = 238, 22 Hz, 1F). ^13^C NMR (75 MHz, CDCl_3_): δ 70.7 (dd, *J* = 26, 19 Hz), 120.0 (dd, *J* = 300, 288 Hz), 123.8, 12.4, 129.8, 130.6, 131.6, 132.6, 132.8, 135.7. Anal. Calcd. for C_14_H_11_BrF_2_O_3_S: C, 44.58; H, 2.94. Found: C, 44.85; H, 3.09. MS (ESI, *m*/*z*): 398.9 (M[^79^Br]+Na^+^), 400.9 (M[^81^Br]+Na^+^). IR (KBr): 3525, 1490, 1451, 1334, 1314, 1159, 1112 cm^-1^.

**2.1.8. 2,2-Difluoro-2-(phenylsulfonyl)-1-[4-(trifluoromethyl)phenyl]ethanol (3h).** White solid, mp 95–96 °C. 36% ee. Ee was determined by HPLC analysis: CHIRALPAK AD-H, hexane:*i*-PrOH = 90:10, flow rate 0.7 mL/min, *t*_major_ = 20.43 min, *t*_minor_ = 21.46 min. ^1^H NMR (300 MHz, CDCl_3_): δ 3.56 (d, *J* = 3.8 Hz, 1H), 5.66 (dt, *J* = 21, 2.7 Hz, 1H), 7.57–7.70 (m, 6H), 7.79 (t, *J* = 7.4 Hz, 1H), 8.00 (d, *J* = 7.8 Hz, 2H). ^19^F NMR (287 MHz, CDCl_3_): δ –63.2(s, 3F), –104.5 (d, *J* = 240 Hz, 1F), –119.6 (dd, *J* = 240, 19.6 Hz, 1F). ^13^C NMR (75 MHz, CDCl_3_): δ 75.7 (dd, *J* = 27, 19 Hz), 120.0 (dd, *J* = 295, 290 Hz), 122.1 (q, *J* = 273 Hz), 125.3 (q, *J* = 3.7 Hz), 128.5, 129.5, 130.6, 131.5, 132.5, 135.8, 137.7. Anal. Calcd. for C_15_H_11_F_5_O_3_S: C, 49.18; H, 3.03. Found: C, 49.16; H, 3.09. MS (ESI, *m*/*z*): 389.0 (M+Na^+^). IR (KBr): 3512, 1453, 1327, 1157, 1112, 1064 cm^-1^.

**2.1.9. 2,2-Difluoro-2-(phenylsulfonyl)-1-*o*-tolylethanol (3i).** White solid, mp 93–95 °C. 11% ee. Ee was determined by HPLC analysis: CHIRALPAK OD, hexane:*i*-PrOH = 98:2, flow rate 0.7 mL/min, *t*_minor_ = 21.68 min, *t*_major_ = 25.85 min. ^1^H NMR (300 MHz, CDCl_3_): δ 2.37 (s, 3H), 3.25 (d, *J* = 3.2 Hz, 1H), 5.93 (dd, *J* = 22, 2.3 Hz, 1H), 7.14–7.31 (m, 3H), 7.62 (m, 3H), 7.77 (t, *J* = 7.5 Hz, 1H), 8.02 (d, *J* = 7.6 Hz, 2H). ^19^F NMR (287 MHz, CDCl_3_): δ –104.9 (d, *J* = 238 Hz, 1F), –120.2 (dd, *J* = 238, 23.5 Hz, 1F). ^13^C NMR (75 MHz, CDCl_3_): δ 19.3 (d, *J* = 2.8 Hz), 66.8 (dd, *J* = 27, 19.6 Hz), 120.7 (dd, *J* = 300, 288 Hz), 126.3, 127.9, 129.2, 129.4, 130.4, 130.7, 132.3, 132.6, 135.6, 136.7. Anal. Calcd. for C_15_H_14_F_2_O_3_S: C, 57.68; H, 4.52. Found: C, 57.64; H, 4.49. MS (ESI, *m*/*z*): 335.0 (M+Na^+^). IR (KBr): 3488, 1452, 1336, 1315, 1154, 1118, 996 cm^-1^.

**2.1.10. 2,2-Difluoro-1-(4-methoxyphenyl)-2-(phenylsulfonyl)ethanol (3j).** ^1^H NMR (300 MHz, CDCl_3_): δ 3.43 (d, *J* = 1.7 Hz, 1H); 3.78 (s, 1H); 5.52 (dt, *J* = 21 Hz, 3.3 Hz, 1H); 6.88 (d, *J* = 8.8 Hz, 2H); 7.39 (d, *J* = 8.8 Hz, 2H); 7.59 (t, *J* = 7.9 Hz, 2H); 7.74 (t, *J* = 7 Hz, 2H); 7.98 (d, *J*=7.5 Hz, 2H). ^19^F NMR (287 MHz, CDCl_3_): δ –103.9 (d, *J* = 239 Hz, 1F); –119.0 (dd, *J* = 239 Hz, *J* = 21 Hz, 1F). The data are consistent with the previous report [5]. 12% ee. Ee was determined by HPLC analysis: CHIRALPAK IC, hexane:*i*-PrOH = 95:5, flow rate 1.0 mL/min, *t*_major_ = 45.51 min, *t*_minor_ = 52.26 min.

**2.1.11. 1-(4-*tert*-Butylphenyl)-2,2-difluoro-2-(phenylsulfonyl)ethanol (3k).** 4% ee. Ee was determined by HPLC analysis: CHIRALPAK IC, hexane:*i*-PrOH = 95:5, flow rate 0.8 mL/min, *t*_minor_ = 18.33 min, *t*_major_ = 21.00 min.

**2.1.12. 2,2-Difluoro-1-(naphthalen-2-yl)-2-(phenylsulfonyl)ethanol (3l).** ^1^H NMR (300 MHz, CDCl_3_): δ 3.67 (s, 1H); 5.77 (d, *J* = 12 Hz, 1H); 7.35–8.15 (m, 12H). ^19^F NMR (287 MHz, CDCl_3_): δ –103.8 (d, 237 Hz, 1F); –121.5 (dd, *J* = 237 Hz, 21 Hz, 1F). The data are consistent with the previous report [6]. [α]^22^_D_ 12.0 (*c* = 0.75, CHCl_3_) (23% ee); ee was determined by HPLC analysis: CHIRALPAK AD-H, hexane:*i*-PrOH = 80:20, flow rate 0.7 mL/min, *t*_minor_ = 23.6 min, *t*_major_ = 26.02 min.

**2.2 Determination of the absolute configuration of (+)-3a.**

To a solution of benzaldehyde (**la**) (255 mg, 2.4 mmol), difluoromethyl phenyl sulfone (**5**) (384 mg, 2.0 mmol) and PTC **6a** (110 mg, 0.20 mmol) in toluene (12 mL) was added powdered KOH portion wise (450 mg, 8.0 mmol) at −20 °C. After the mixture was stirred at −20 °C for 48 h, the reaction was quenched with 1N HCl (12 mL), and the mixture was extracted with ethyl acetate (20 mL x 3). The organic extracts were washed with brine and dried over MgSO_4_. Removal of the solvent followed by flash colunm chromatography of the residue (silica gel, hexane:AcOEt = 6:1) gave **3a** as a colorless solid (575 mg, 96%, 44% ee).

Into a 50 mL Schlenk flask containing a solution of (+)-**3a (**298 mg, 1.0 mmol**)** in 11 mL DMF at room temperature was added 6 mL of a HOAc-NaOAc (1:1) buffer solution (8 mol/L). Magnesium turnings (360 mg, 15 mmol) were then added portion wise. The reaction mixture was stirred at room temperature for 3 h, followed by adding 30 mL water. The reaction mixture was extracted with Et_2_O (20 mL x 3), and the combined organic phase was washed with saturated aqueous NaHCO_3_ and brine, then dried over MgSO_4_. After the removal of the solvent, the crude product was purified by silica gel column chromatography using petroleum ether-ethyl acetate (6 : 1) as the eluent to give product **10** as a colorless oil, 148 mg (93% yield). ^1^H NMR (300 MHz, CDCl_3_): δ 2.56 (d, *J* = 4 Hz, 1H), 4.82 (m, 1H), 5.76 (td, *J* = 56, 4.7 Hz, 1H), 7.35–7.43 (m, 5H). ^19^F NMR (287 MHz, CDCl_3_): δ –127.4 (ddd, *J* = 283, 56, 10 Hz, 1F), –128.6 (ddd, *J* = 283, 56, 11.5 Hz, 1F). The data are consistent with the previous report [6]. [α]^22^_D_ 7.8 (*c* = 0.6, CH_2_Cl_2_) (43% ee); ee was determined by HPLC analysis: CHIRALPAK IC, hexane:*i*-PrOH = 95:5, flow rate 0.8 mL/min, *t*_minor_ = 6.68 min, *t*_major_ = 7.38 min.

By comparing the rotation of our synthetic **9** with that reported for (*R*)-**9** ([α]^24^_D_ –11.5 (*c* = 1.0, CH_2_Cl_2_)) [7], we concluded that the configuration of (+)-**3a** is *S*.

**2.3 Determination of the absolute configuration of (+)-3b**–**h and (+)-3l** [8]**.**

As a typical example, determination of the absolute configuration of **3b**: To a solution of (R)-MTPA chloride in 0.3 mL anhydrous CH_2_Cl_2_ was added 3 mg of (+)-**3a**, 0.015 mL Et_3_N and one crystal of DMAP. After 2 h of stirring, direct ^19^F NMR analysis was carried out. ^19^F NMR δ(CF_3_)_major_ –72.4, δ(CF_3_)_minor_ –72.8.

(+)-**3b** ^19^F NMR δ(CF_3_)_major_ –72.4, δ(CF_3_)_minor_ –72.9.

By comparing the Mosher's ester’s chemical shift of the main isomer, (+)-**3b** should have the same configuration as (+)-**3a**.

**References**

1. Caron, S.; Do, N. M.; Arpin, P.; Larivée, A. *Synthesis* **2003,** *11,* 1693–1698. doi:[10.1055/s-2003-40889](http://dx.doi.org/10.1055/s-2003-40889)
2. Arai, S.; Ishida, T.; Shioiri, T. *Tetrahedron Lett.* **1998,** *39,* 8299–8302. doi:[10.1016/S0040-4039(98)01811-5](http://dx.doi.org/10.1016/S0040-4039(98)01811-5)
3. Arai, S.; Tsuge, H.; Oku, M.; Miura, M.; Shioiri, T. *Tetrahedron* **2002,** *58,* 1623–1630. doi:[10.1016/S0040-4020(02)00028-5](http://dx.doi.org/10.1016/S0040-4020(02)00028-5)
4. Ku, J.-M.; Yoo, M.-S.; Park, H.; Jew, S.; Jeong, B.-S. *Tetrahedron* **2007,** *63,* 8099–8103. doi:[10.1016/j.tet.2007.06.012](http://dx.doi.org/10.1016/j.tet.2007.06.012)
5. Stahly, G. P. *J. Fluorine Chem.* **1989,** *43,* 53–66. doi:[10.1016/S0022-1139(00)81636-X](http://dx.doi.org/10.1016/S0022-1139(00)81636-X)
6. Prakash, G. K. S.; Hu, J.; Wang, Y.; Olah, G. A. *Eur. J. Org. Chem.* **2005,** 2218–2223. doi:[10.1002/ejoc.200500101](http://dx.doi.org/10.1002/ejoc.200500101)
7. Matsuda, T.; Harada, T.; Nakajima, N.; Itoh, T.; Nakamura, K. *J. Org. Chem.* **2000,** *65,* 157–163. doi:[10.1021/jo991283k](http://dx.doi.org/10.1021/jo991283k)
8. Gaspar, J.; Guerrero, A. *Tetrahedron: Asymmetry* **1995,** *6,* 231–238. doi:[10.1016/0957-4166(94)00379-P](http://dx.doi.org/10.1016/0957-4166(94)00379-P)
